# Coexpression of CCR7 and CXCR4 During B Cell Development Controls CXCR4 Responsiveness and Bone Marrow Homing

**DOI:** 10.3389/fimmu.2019.02970

**Published:** 2019-12-18

**Authors:** Saria Mcheik, Nils Van Eeckhout, Cédric De Poorter, Céline Galés, Marc Parmentier, Jean-Yves Springael

**Affiliations:** ^1^Institut de Recherche Interdisciplinaire en Biologie Humaine et Moléculaire (IRIBHM), Université Libre de Bruxelles (ULB), Campus Erasme, Brussels, Belgium; ^2^Institut des Maladies Métaboliques et Cardiovasculaires, Institut National de la Santé et de la Recherche Médicale, Université Toulouse III Paul Sabatier, Toulouse, France; ^3^Walloon Excellence in Life Sciences and Biotechnology, Brussels, Belgium

**Keywords:** B cells, homing, lymphopoiesis, CXCR4, CCR7

## Abstract

The CXCL12–CXCR4 axis plays a key role in the retention of stem cells and progenitors in dedicated bone marrow niches. It is well-known that CXCR4 responsiveness in B lymphocytes decreases dramatically during the final stages of their development in the bone marrow. However, the molecular mechanism underlying this regulation and whether it plays a role in B-cell homeostasis remain unknown. In the present study, we show that the differentiation of pre-B cells into immature and mature B cells is accompanied by modifications to the relative expression of chemokine receptors, with a two-fold downregulation of CXCR4 and upregulation of CCR7. We demonstrate that expression of CCR7 in B cells is involved in the selective inactivation of CXCR4, and that mature B cells from CCR7^−/−^ mice display higher responsiveness to CXCL12 and improved retention in the bone marrow. We also provide molecular evidence supporting a model in which upregulation of CCR7 favors the formation of CXCR4–CCR7 heteromers, wherein CXCR4 is selectively impaired in its ability to activate certain G-protein complexes. Collectively, our results demonstrate that CCR7 behaves as a novel selective endogenous allosteric modulator of CXCR4.

## Introduction

The retention of hematopoietic stem cells and progenitors in the bone marrow (BM) is a complex process that depends on chemoattraction and adhesion signals (1). Among these signals, the CXCL12 chemokine has been identified as a key player controlling the homing of stem cells, their retention in dedicated BM niches and the proliferation of human and mouse progenitor cells. Several studies have also indicated that CXCL12 plays an important role in hematologic malignancies by conferring cell survival and protection against cytotoxic therapies (2). In BM, CXCL12 is expressed at high levels by several cell types, including osteoblasts, endothelial cells and a subset of reticular cells speckled in the marrow (3–5). CXCL12 binds to the chemokine receptor, CXCR4, which couples to pertussis toxin-sensitive Gi/o proteins and activates downstream signals such as cAMP inhibition, calcium mobilization, MAPK phosphorylation and chemotaxis. In regard to B-cell lymphopoiesis, the CXCL12–CXCR4 axis has been described as a major player in the homeostasis of B-cell precursors in the BM (6–9). Deficiency in CXCL12 or CXCR4 leads to a decrease in cell retention in the BM and the mobilization of B-cell precursors in the blood circulation (10, 11). Similarly, inhibition of the CXCR4 receptor by the CXCR4-selective antagonist, Plerixafor/AMD3100, induces the recruitment of progenitors to the peripheral blood (12). It has been known that when lymphopoiesis progresses to more differentiated stages, B cells lose their responsiveness to CXCL12 despite the continuous expression of the CXCR4 receptor (7, 13–16). Nonetheless, B cells regain their sensitivity to CXCL12 as they further differentiate into plasma cells; however the mechanism involved in this transient loss of CXCR4 responsiveness has not been identified. B-cell differentiation in the BM is accompanied by modulation of the expression of several receptors. Among these, CCR7 is strongly upregulated during the differentiation of pre-B cells to the immature and mature stages (17). CCR7 is required, together with CXCR4 and CXCR5, for B cells to enter lymphoid organs that express high levels of the chemokines CCL19 and CCL21. Since CCR7 upregulation takes place in B-cell populations known to display poor CXCR4 responsiveness, we investigated whether CCR7 may be involved in the inhibition of CXCR4 function.

In the present study, we identify a novel regulatory function associated with CCR7 expression, showing that CCR7 controls the responsiveness of CXCR4 and the homing of B cells into the BM parenchyma. Using a combination of approaches, we demonstrate that CCR7 physically interacts with CXCR4 and that CCR7–CXCR4 heteromers exhibit reduced CXCR4 signaling capacity as compared with CXCR4 homomers.

## Materials and Methods

### Mouse Models

The CCR7-deficient mouse line, described previously (18), was obtained from The Jackson Laboratory and backcrossed on the C57BL/6 background for at least eight generations. The Boy/J (CD45.1^+^) mouse line was obtained from Janvier laboratory. All mice were housed under specific-pathogen-free conditions at our local mouse facility. In all experiments, CCR7-deficient mice (6–8 weeks old) were compared with wild-type littermates resulting from CCR7^+/−^ intercrossing. To generate BM chimeras, recipient mice were irradiated lethally (10 Gy) and their immune system was reconstituted by intravenous injection of 2 10^6^ total BM cells from donor mice. Chimera were analyzed 8–10 weeks after reconstitution. Animal experimentation was carried out in accordance with European (EU Directives 86/609/EEC) and national guidelines. All procedures were reviewed and approved by the local ethical committee (Commission d'Ethique du Bien-Etre Animal, CEBEA) of the Université Libre de Bruxelles.

### Expression of Recombinant Receptors and Mutants

All the receptors used in this study were either cloned into bicistronic pEFIN3 vector (Euroscreen) for stable expression or pcDNA3 vector (Life Technologies) for transient expression (Table S1). All constructs were verified by sequencing prior to transfection.

### Cell Culture and Transfections

The human pre-B cell line Nalm-6 (ACC-128, DMSZ) was cultured in RPMI 1640 containing L-glutamine supplemented with 10% heat-inactivated FBS, 100 U/ml penicillin, 100 μg/ml streptomycin and 50 mM 2-mercaptoethanol (Sigma). Transfection of Nalm-6 cells by pEFIN3 plasmids was performed by electroporation (0.3 kV, 960 μF) and cells stably expressing the receptors of interest were selected and cultured in the presence of 800 μg/ml G418 (Invitrogen). The human embryonic kidney cell line HEK293T (CRL-3216, ATCC) was cultured in Dulbecco's Modified Eagle's Medium (DMEM) supplemented with 10% FBS (GIBCO), 100 U/ml penicillin and 100 μg/ml streptomycin (Invitrogen). Transfection of HEK293T cells by pcDNA3 plasmids was performed 24 h after cell seeding using the calcium phosphate precipitation method.

### Flow Cytometry

Mononuclear cells from blood, BM, spleen or lymph nodes were isolated by Ficoll-Paque separation, diluted in staining buffer (PBS with 0.1% BSA and 0.01% sodium azide) and incubated with 1 μg rat anti-mouse FcγR antibody (Clone 2.4G2, BD Biosciences) to prevent binding of conjugated antibodies to FcγR. Cells were further incubated for an additional 30 min on ice with a mixture of the following antibodies: Alexa Fluor 700 rat anti-mouse CD45R (B220, clone RA3-6B2, BD Biosciences), FITC rat anti-mouse CD43 (Clone S7, BD Biosciences), PerCP-Cy5.5 rat anti-mouse IgM (clone R6-60.2, BD Biosciences), and V450 rat anti-mouse IgD (clone 11-26c.2a, BD Biosciences). This staining strategy allowed to distinguish the developmental subsets of B cells, namely pre-B cells (B220^+^/CD43^−^/IgM^−^/IgD^−^), immature B cells (B220^+^/CD43^−^/IgM^+^/IgD^−^) and mature B cells (B220^+^/CD43^−^/IgM^+^/IgD^+^) (19, 20). As an alternative staining strategy, cells were incubated with a mixture of Alexa Fluor 700 rat anti-mouse CD45R, PerCP-Cy5.5 rat anti-mouse IgM, PE rat anti-mouse AA4.1 (clone AA4.1, BD Biosciences) and FITC rat anti-mouse CD24/HSA (clone M1/69, BD Biosciences) to discriminate pre-B cells (B220^+^/IgM^−^/AA4.1^+^/HSA^+^), immature B cells (B220^+^/IgM^+^/ AA4.1^+^/HSA^+^) and mature B cells (B220^+^/CD43^+^/AA4.1^low^/HSA^low^) (21). Absolute cell numbers were determined by incorporating beads in the cell suspension (15 μm, Bangs Laboratories) and acquiring 15,000 beads. Expression of chemokine receptors on B cell subsets was estimated by using the following antibodies: phycoerythrin anti-mouse CXCR4 (clone 2B11, eBioscience), allophycocyanin anti-mouse CCR7 (clone 4B12, eBioscience), allophycocyanin anti-mouse CXCR5 (clone 614641, R&D Systems) and allophycocyanin rat anti-mouse CCR6 (clone 140706, R&D Systems).

### RNA Extraction and RT-qPCR

RNA was extracted from sorted B cells subpopulations using the RNeasy kit (Qiagen) according to the manufacturer's instructions. Quantification of gene expression was performed by using the KAPA SYBR-FAST One-Step qRT-PCR kit and the CFX-Connect Real-time PCR detection system (Bio-Rad Laboratories) following the manufacturer's instructions. The following primers were used: CXCR4 (sense: 5′-AAGAAGTGGGTTCTGGAGAC-3′, anti-sense: 5′-GACTATGCCAGTCAAGAAG-3′), CCR7 (sense: 5′-CCTGCCTCTCATGTATTCTG-3′, anti-sense: 5′-GGTTGAGCAGGTAGGTATCC-3′), CXCR5 (sense: 5′-ATTTTCTTCCTCTGCTGGTC-3′, anti-sense 5′-GAATTCACACAAGGTGATGG-3′), CCR6 (sense: 5′-AGATCATGAAGGATGTGTGG-3′, anti-sense: 5′- TACATGGTAAAGGACGATGC-3′), Beta-actin (sense: 5′-CAGCTTCTTTGCAGCTCCTT-3′, anti-sense: 5′-CACGATGGAGGGGAATACAG-3′) and GAPDH (sense: 5′-AAGGGCTCATGATGACCACAGTC-3′, anti-sense: 5′-CAGGGATGATGTTCTGGGCA-3′).

### Chemotaxis Assay

The migration of splenic and BM B cell populations in response to chemokine gradients was performed in 6-well Costar transwell chambers (5 μm pore size, Corning). Briefly, a suspension of 10^6^ mononuclear cells in RPMI 1640 supplemented with 10% FCS was added to each insert in a well containing a solution of chemokine (R&D Systems). In some experiments, blocking anti-CXCR4 MAB21651 (247506, R&D Systems) or anti-CCR7 MAB3477 (4B12, R&D Systems) antibodies were added to the cells prior to migration. Wells containing medium without chemokines were used as controls. After 2 h at 37°C, cells in the bottom of the wells were harvested, diluted in FACS staining buffer and incubated with antibodies and counting beads to discriminate and quantify B cell subsets. The migration of Nalm-6 cells was performed in 96-well Costar transwell chambers (5 μm pore size, Corning NY). A cell suspension of 10^4^ Nalm-6 in RPMI 1640 supplemented with 10% FCS was added to each insert. Wells containing medium without chemokines were used as controls. After 1 h at 37°C, cells in the bottom of the wells were counted by using the ATPlite luminescence assay kit (PerkinElmer, Waltham, MA). The results are expressed as chemotaxis index, i.e., the ratio of cells migrating in response to the chemoattractant over cells migrating toward the medium alone.

### Cell Adhesion Assay

Short-term adhesion assays were performed as previously described (8). Briefly, 2.10^4^ Nalm-6 cells in suspension in PBS supplemented with 0.1% BSA were stimulated with 300 nM CXCL12 for 1 min and then added to VCAM-1-coated wells (with a 1 mg/ml solution). Cells were quickly spun down and allowed to settle for another minute at 37°C. As controls, cells unstimulated with CXCL12 and uncoated wells were used. Wells were then washed twice and the number of adherent cells was determined using the ATPlite luminescence assay kit (PerkinElmer, Waltham, MA).

### B Cell Homing Assay

B220^+^ B cells were recovered from BM mononuclear cells from WT or CCR7^−/−^ mice by negative selection using a MACS microbeads isolation kit (Miltenyi Biotec) according to the manufacturer's instructions. Then, 5.10^6^ cells were incubated for 15 min in PBS containing 5 μM CFDA succinimidyl ester (Molecular Probes). After washing, about 2.10^6^ CFDA-stained B220^+^ cells were injected retro-orbitally into 6- to 8-week-old syngenic C57BL/6 recipient mice. The percentage of CFDA-labeled cells in the cell mixture was determined by flow cytometry before injection to know the number of CFDA-labeled cells that are transferred. Recipient mice were killed 2 h after injection and BM B cell populations (CFDA-labeled or not) were analyzed by FACS.

### Bi-Molecular Complementation Assay

Bi-molecular fluorescence and luminescence complementation assays were performed in HEK293T cells as described previously (22). Briefly, plasmids expressing the various receptors fused to split mVenus or Rluc8 fragments were transfected into HEK293T cells. A control corresponding to mock-transfected cells was included in order to subtract raw basal luminescence and fluorescence from the data. Forty-eight hours after transfection, cells were washed twice with PBS, detached and resuspended in PBS. Approximately 2.10^5^ cells were distributed per well of 96-well plates. The 535 nm fluorescence following excitation at 485 nm, and the luminescence after incubation with 5 μM coelenterazine H (Promega) were recorded using a Mithras LB940 reader (Berthold).

### BRET Proximity Assay

BRET proximity assays were performed as described previously (23). Briefly, HEK-293T cells were transfected using a constant amount of plasmid DNA but various ratios of plasmids encoding the fusion protein partners. A control corresponding to mock-transfected cells was included in order to subtract raw basal luminescence or fluorescence from the data. Expression of mVenus fusion proteins was estimated by measuring fluorescence at 535 nm following excitation at 485 nm. Expression of RLuc fusion proteins was estimated by measuring the luminescence of the cells after incubation with 5 μM coelenterazine H (Promega). In parallel, BRET^1^ between h*R*Luc8 and mVenus was measured 5 min after addition of 5 μM coelenterazine H (Promega). BRET^1^ readings were collected using a Mithras LB940 reader (Berthold). The BRET^1^ signal was calculated as the ratio of emission of mVenus (510–590 nm) to h*R*Luc8 (440–500 nm)—Cf, where Cf corresponds to the ratio of emission (510–590 nm) to (440–500 nm) for the hRLuc construct expressed alone in the same experiment.

### G Protein BRET Assay

G protein activation was assayed by BRET as previously described (24). Briefly, plasmids encoding G protein biosensors and receptors of interest were cotransfected into HEK293T cells. Forty-eight hours after transfection, cells were washed twice with PBS, detached and resuspended in PBS containing 0.1% (w/v) glucose at room temperature. Cells were then distributed (80 μg of proteins per well) in a 96-well microplate (Optiplate, PerkinElmer). BRET^2^ between *R*Luc8 and GFP10 was measured 1 min after addition of 5 μM coelenterazine 400a/Deep blue C (Gentaur). BRET readings were collected using an Infinite F200 reader (Tecan). The BRET signal was calculated as the ratio of emission of GFP10 (510–540 nm) to *R*Luc8 (370–450 nm).

### Statistical Analyses

Results are expressed as arithmetic means ± SEM. Significance was determined using Tukey's test and the Prism4 software (GraphPad). For all tests, values of *p* < 0.05 were considered as significant.

## Results

### CCR7 Inhibits CXCR4 Responsiveness During B-Cell Development

**Figure 1:**
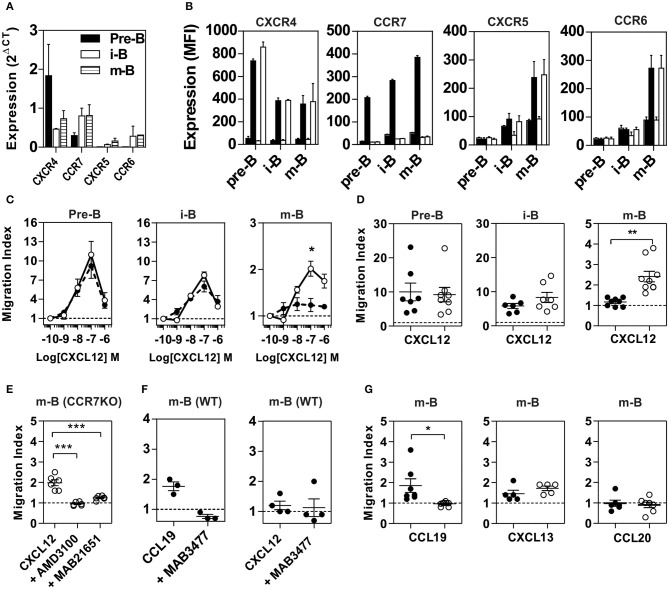
Properties of B cell populations prepared from CCR7^+/+^ or CCR7^−/−^ mice. **(A)** Expression of chemokine receptors in B cell subpopulations. BM B cell subpopulations were discriminated and sorted by flow cytometry and the expression of CXCR4, CCR7, CXCR5, and CCR6 was quantified by RT-qPCR using GAPDH and β-actin as references. **(B)** Cell surface expression of chemokine receptors. The cell surface expression of chemokine receptors in B cell subpopulations was estimated by flow cytometry using PE-conjugated anti-CXCR4 and APC-conjugated anti-CCR7, anti-CXCR5, and anti-CCR6 antibodies. Bars represent mean values ± SEM (*n* = 5) of the mean fluorescence index, detected in Pre-B, immature (i-B) or mature B cells (m-B) from CCR7^+/+^ (black bars) or CCR7^−/−^ mice (white bars). The first black and white bar represents the mean fluorescence of control isotype. **(C)** Chemotaxis of BM B cells toward CXCL12. Transwell migration of BM B cells from CCR7^+/+^ (black dots) or CCR7^−/−^ (white dots) mice in response to increasing concentrations of CXCL12. Migration index after a 2 h incubation were plotted for each subpopulation. All conditions were run in triplicates and the data, representative of two independent experiments, are presented as mean values ± SEM, ^*^P < 0.05). **(D)** Enhanced chemotactic response to CXCL12 in mature B cells from CCR7^−/−^ mice. Transwell migration of BM B cells from CCR7^+/+^ (black dots) and CCR7^−/−^ (white dots) mice in response to 100 nM CXCL12. Migration indexes were plotted for each subpopulation. Data are represented as mean values ± SEM and dots correspond to individual mice (*n* = 6–8; ^**^P < 0.005). **(E)** Blockade of CXCR4 inhibits CXCL12-elicited migration of CCR7^−/−^ mature B cells. Transwell migration of BM mature B cells from CCR7^−/−^ mice in response to 100 nM CXCL12 in the presence or not of 1 μM AMD3100 or 5 μg/ml MAB2165 (blocking anti-CXCR4 monoclonal). Data are presented as mean values ± SEM and dots correspond to individual mice (*n* = 6; ^***^P < 0.0005). **(F)** Blockade of CCR7 does not affect CXCR4 responsiveness in CCR7^+/+^ mature B cells. Transwell migration of BM mature B cells from CCR7^+/+^ mice in response to 100 nM CCL19 or CXCL12, and in the presence or not of 5 μg/ml MAB3477 (blocking anti-CCR7 monoclonal). Data are presented as mean values ± SEM, and dots correspond to individual mice **(G)** CCR7-deficiency abrogates the CCL19-dependent migration of mature B cells but does not affect the migration in response to CXCL13 or CCL20. Transwell migration of BM mature B cells from CCR7^+/+^ (black dots) and CCR7^−/−^ (white dots) mice in response to 100 nM CCL19, CXCL13, or CCL20. Data are represented as mean values ± SEM, and dots correspond to individual mice (*n* = 5 to 8; ^*^P < 0.05).

To evaluate the influence of CCR7 on CXCR4 function, we first tested the expression and functional response of the two receptors in various B-cell populations from WT and CCR7^−/−^ mice. BM cells were sorted into three subpopulations according to established markers, and the expression of chemokine receptors was measured by RT-qPCR (19, 20) (Figure 1A and Figure S1). In agreement with previous studies, we show that CXCR4 was expressed in pre-B cells and that its expression was decreased by ~3-fold in immature and mature B cells. In contrast, the expression of CCR7 was weak in pre-B cells and increased by ~2-fold as differentiation progressed to immature and mature B cells. Finally, the expression of CXCR5 and CCR6 was barely detectable in pre-B cells but was increased in immature and mature B cells. Using FACS, we confirm that CXCR4 was expressed at the surface of pre-B cells and that its expression was decreased in immature and mature B cells (Figure 1B). In comparison, the cell surface expression of CCR7 was weak in pre-B cells but increased as differentiation progressed to the immature and mature stages. In agreement with the RT-qPCR data, the cell-surface expression of CXCR5 and CCR6 was only detectable in immature and mature B cells (Figure 1B). Since CCR7 upregulation at the cell surface takes place in populations known to display poor responsiveness to CXCR4 agonists, we questioned whether it may be involved in the impairment of CXCR4 activity. We first investigated the impact of CCR7 expression on the presence of CXCR4 at the cell surface, and showed that the signal for CXCR4, as well as for CCR6 and CXCR5, was similar in populations from CCR7^−/−^ and control mice (Figure 1B). Subsequently, we investigated the impact of CCR7-deficiency on the responsiveness of CXCR4 by measuring the ability of B cells to migrate *ex vivo* toward a CXCL12 gradient. In agreement with previous studies (13–17), the chemotaxis of B cells from CCR7^+/+^ control mice decreased as differentiation progressed, with the mature B cells being almost unresponsive to CXCL12 (Figures 1C,D and Figure S2). In contrast, mature B cells from CCR7^−/−^ mice migrated significantly more efficiently than control cells (Figures 1C,D and Figures S1, S2). A higher migration index was also observed in immature B cells from CCR7^−/−^ mice, although the difference did not reach statistical significance. The migration of CCR7-deficient mature B cells was completely abrogated upon pre-treatment with the CXCR4-selective antagonist, AMD3100, or the blocking monoclonal antibody, MAB21625, confirming the involvement of CXCR4 (Figure 1E). In contrast, CCR7 blockade by the monoclonal antibody, MAB3477, did not restore CXCR4 responsiveness to CCR7^+/+^ mature B cells, indicating that CCR7 signaling is not required (Figure 1F). Importantly, CCR7-deficiency did not increase the responsiveness of CXCR5 or CCR6, suggesting that CCR7 selectively controls the function of CXCR4 (Figure 1G).

### CCR7 Regulates the Number of Mature B Cells in the BM

**Figure 2:**
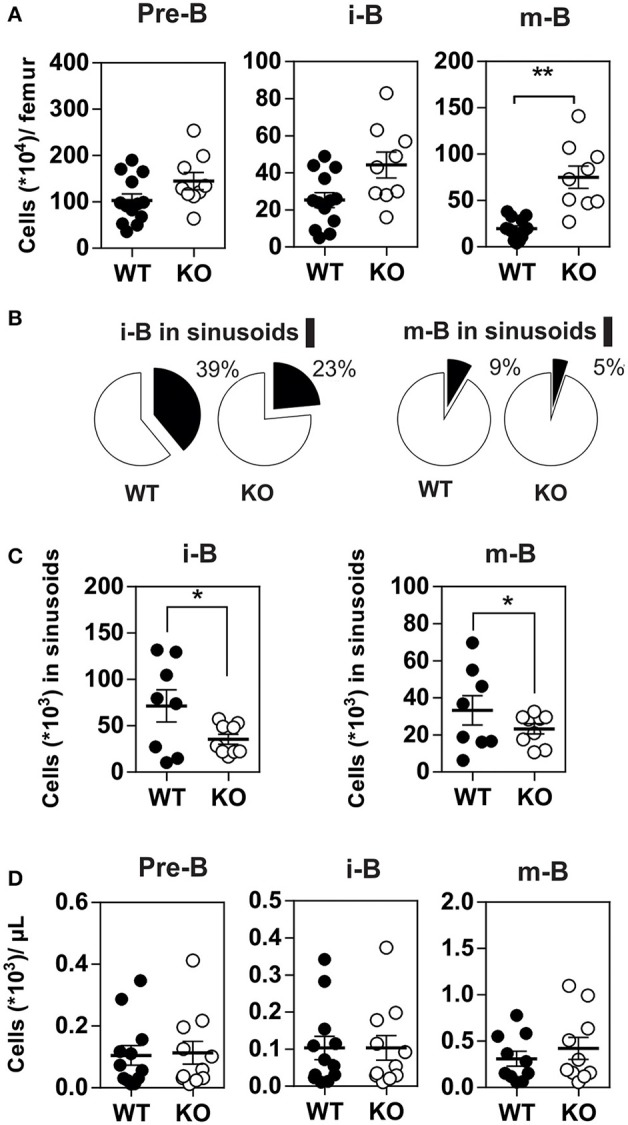
**(A)** Increased number of mature B cells in the BM of CCR7^−/−^ mice. The number of B cell subpopulations was determined in the BM of CCR7^+/+^ (black dots) and CCR7^−/−^ (white dots) mice. Data are represented as mean values ± SEM and dots correspond to individual mice (*n* = 10 to 13 mice; ^*^P < 0.05; ^**^P < 0.005). **(B,C)** Decreased egress of immature and mature B cells in the BM sinusoids of CCR7^−/−^ mice. *In vivo* labeling of BM B cell subsets by injection of PE-conjugated anti-CD19 antibodies 4 min before sacrifice and tissue collection. The proportions of immature and mature B cells in the parenchyma (CD19^−^; white) and sinusoids (CD19^+^; black) are displayed in **(B)** and the number of immature and mature B cells in sinusoids displayed in **(C)**. Data for CCR7^+/+^ (black dots) and CCR7^−/−^ (white dots) mice are represented as mean values ± SEM and dots correspond to individual mice (*n* = 8–9 mice; ^*^P < 0.05). **(D)** B cell counts are comparable in the blood of CCR7^+/+^ and CCR7^−/−^ mice. The number of B cell subpopulations was determined in the blood of CCR7^+/+^ (black dots) and CCR7^−/−^ mice (white dots). Data are represented as mean values ± SEM and dots correspond to individual mice (*n* = 10–13 mice; ^*^P < 0.05; ^**^P < 0.005).

**Figure 3:**
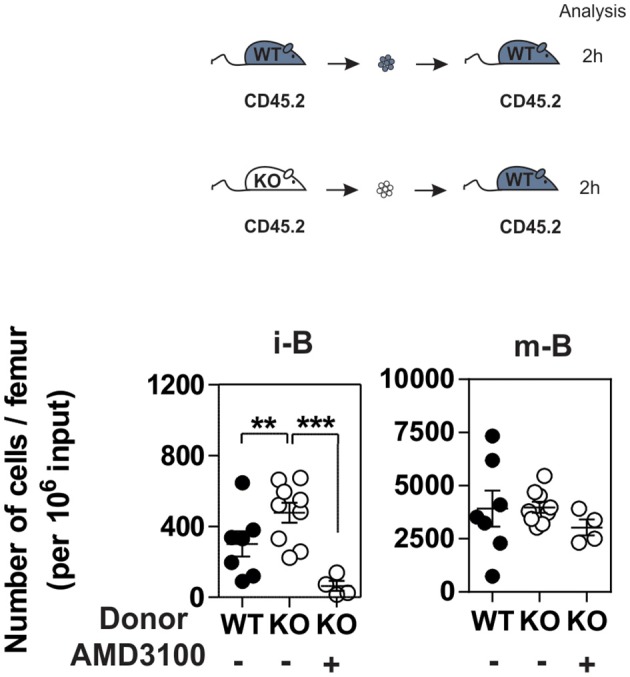
CCR7 deficiency results in increased homing of immature B cells to BM. B220^+^ cells purified from BM of CCR7^+/+^ (black dots) or CCR7^−/−^ mice (white dots) were labeled with CFDA-SE and transferred into wild type recipients by injection into the retro-orbital venous plexus in combination or not with AMD3100. Two hours later, BM mononuclear cells were isolated and B cell subpopulations were analyzed by flow cytometry. Data are represented as mean values ± SEM and dots correspond to individual mice (*n* = 4–8 mice; ^**^P < 0.005; ^***^P < 0.0005).

**Figure 4:**
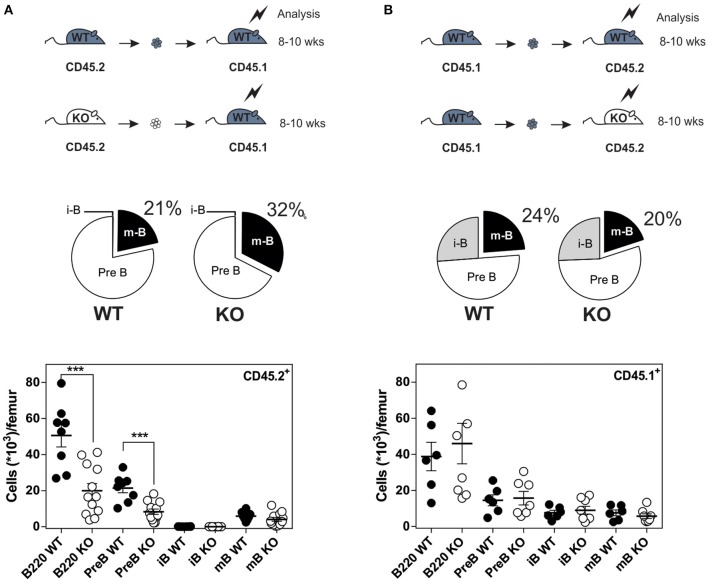
Increased number of B cells in the BM of CCR7^−/−^ mice is not due to an alteration of BM environment. **(A)** Distribution and quantification of donor CD45.2^+^ (WT or CCR7^−/−^) B cells recovered from the BM of chimeric CD45.1^+^ recipients. **(B)** Distribution and quantification of donor CD45.1^+^ WT B cells recovered from the BM of chimeric CD45.2^+^ (WT or CCR7^−/−^) recipients. Data are represented as mean values ± SEM and dots correspond to individual mice (*n* = 8–12 mice; ^***^P < 0.005).

Since CXCR4 plays a major role in the homing of progenitors to the BM, we measured the number of B cells in mice expressing or lacking CCR7, and found that the total number of B220^+^ B cells was increased moderately in the BM of CCR7^−/−^ mice (Figure S3). Analysis of B-cell subpopulations shows that the number of mature B cells was increased in the BM of CCR7^−/−^ mice (Figure 2A and Figure S4), while the number of CD4^+^ and CD8^+^ cells remained similar in CCR7^−/−^ and wild type mice (Figure S5). Next, we performed an *In vivo* pulse-labeling experiment to discriminate B-cell subpopulations present in the BM parenchyma and sinusoids (25). Mice were injected with a phycoerythrin-conjugated CD19 antibody shortly before sacrifice, and B-cell subpopulations in sinusoids (CD19^+^) and parenchyma (CD19^−^) were evaluated. Analysis shows that the proportion and number of immature and mature B cells were reduced in the sinusoids of CCR7^−/−^ mice (Figures 2B,C). Yet, the number of immature and mature B cells in the blood was similar in CCR7^−/−^ and wild type mice, which is in agreement with previous studies (Figure 2D) (18). Collectively, these data indicate that the regulation of CXCR4 responsiveness by CCR7 control the number of immature and mature B cells present in the BM sinusoid. To further analyze the contribution of CCR7 to B-cell homing, we performed an adoptive transfer experiment, comparing the ability of B cells to migrate and home to the BM by fluorescently labeling WT and CCR7^−/−^ B220^+^ cells and transferring them into WT mice. Experiments performed in the presence of the CXCR4-selective antagonist, AMD3100, reveal that CXCR4 was required for the short-term homing of immature but not mature B cells in the BM. CCR7-deficiency increased the homing of transferred immature B cells to the BM by ~2-fold (Figure 3). In contrast, mature B cells populated the BM with similar efficiency irrespective of CCR7 expression, suggesting that the signals required for the short-term homing of mature B cells are not sensitive to CCR7. Subsequently, we used BM cells from CCR7^−/−^ or WT CD45.2^+^ mice to reconstitute the hematopoietic compartment of irradiated WT CD45.1^+^ mice (Figure 4A), showing that the number of CCR7^−/−^ B220^+^ cells was reduced in the BM of reconstituted mice. Analysis of B-cell subpopulations showed that the number of mature B cells remained unaffected, while the number of pre-B cells decreased significantly, resulting in a higher proportion of CCR7^−/−^ mature B cells in the BM of reconstituted mice. However, whether this observation is due to a more efficient retention of mature B cells or a direct effect of CCR7 on hematopoiesis remains to be determined. We also generated reverse chimeras in which CCR7^−/−^ and WT CD45.2^+^ mice were irradiated and reconstituted with the BM of WT CD45.1^+^ mice (Figure 4B). In this setting, the number of B cells remained similar in both groups, indicating that the increased retention of B cells in the BM of CCR7^−/−^ mice is not due to an alteration in the BM microenvironment.

### Regulation of CXCR4 Responsiveness Does Not Require CCR7 Signaling

**Figure 5:**
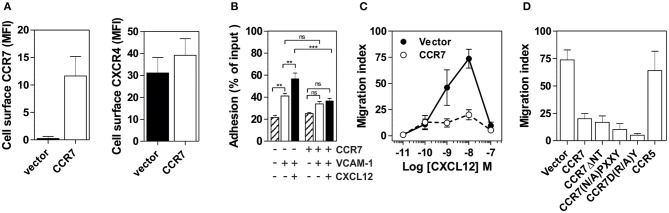
**(A)** Expression of CCR7 in Nalm-6 does not inhibit CXCR4 expression. Nalm-6 cells were stably transfected with a CCR7-encoding plasmid and cell surface expression of CXCR4 and CCR7 was monitored by flow cytometry. The data represent mean values of the mean fluorescent index ± SEM (*n* = 3). **(B)** Expression of CCR7 in Nalm-6 cells inhibits CXCL12-induced adhesion to VCAM-1. A suspension of Nalm-6 cells expressing CCR7 or not were stimulated with 1 μM CXCL12 for 1 min (black bars) or incubated in buffer (white bars) and then allowed to settle in VCAM-1-coated wells for 1 min. Uncoated wells were used as controls (dashed bars). Non-adherent cells were subsequently washed away and adherent cells were counted relative to the number of input cells. The data represent mean values ± SEM (*n* = 3; ^**^P < 0.05; ^***^P < 0.005). **(C)** Expression of CCR7 in Nalm-6 cells inhibits CXCL12-induced chemotaxis. Migration of Nalm-6 cells expressing CCR7 (white dots) or not (black dots) was recorded in Transwells in response to increasing concentrations of CXCL12. The data represent mean values ± SEM (*n* = 3). **(D)** Expression of non-functional CCR7 mutants also inhibits CXCR4 responsiveness. Transwell migration of Nalm-6 cells stably expressing wild-type or non-functional CCR7 mutants was assayed in response to 10 nM CXCL12. Cells stably expressing CCR5 were used as control. The data represent mean values ± SEM (*n* = 3).

To obtain further insight into the mechanisms underlying the inhibition of CXCR4 responsiveness in B cells, we overexpressed CCR7 in the human pre-B cell line Nalm-6. While expression of CCR7 did not affect the presence of CXCR4 at the cell surface (Figure 5A), it induced a strong inhibition of CXCR4 responsiveness as measured by cell adhesion and chemotaxis assays (Figures 5B–D). In comparison, the expression of CCR5 did not impact CXCR4 function (Figure 5D). Thus, expression of CCR7 in a human pre-B cell line appears to be sufficient to mimic the inhibition of CXCR4 responsiveness detected during normal B-cell development *In vivo*. Subsequently, we took advantage of this expression system to investigate the effects of CCR7 mutants unable to bind chemokines (CCR7^ΔNT^) or to activate signaling pathways (CCR7^(N/A)PXXY^ and CCR7^D(R/A)Y^). The expression of the three non-functional CCR7 mutants did not modify the presence of CXCR4 at the cell surface; however, they inhibit CXCR4 responsiveness as efficiently as wild type CCR7 (Figure 5D and Figure S6), confirming that signaling of CCR7 is not required for its inhibitory activity on CXCR4 function.

### CCR7 Inhibits CXCR4-Mediated Gαi Protein Activation

**Figure 6:**
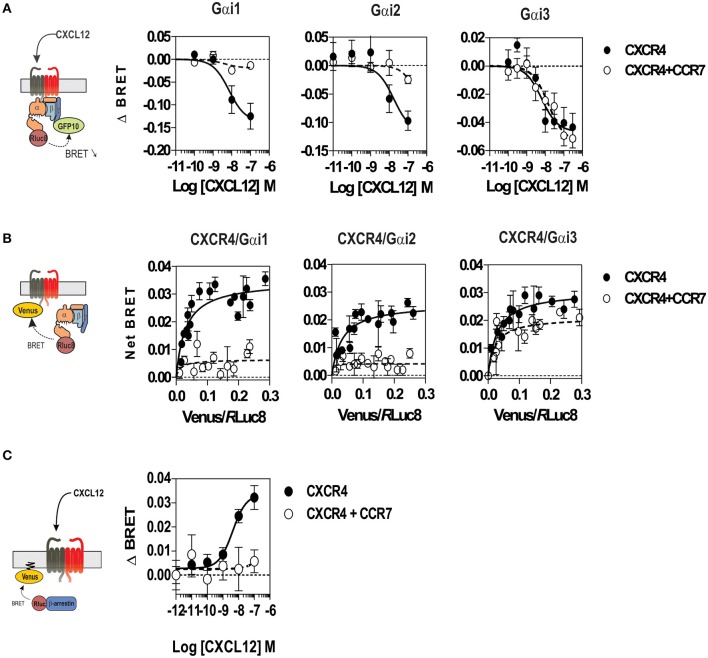
**(A)** CCR7 inhibits the CXCL12-induced activation of Gαi1 and Gαi2 proteins. Real-time measurement of BRET signal in HEK293T cells coexpressing Gαi1, Gαi2, or Gαi3 biosensors with CXCR4 only (black dots) or in a combination with CCR7 (white dots). Cells were stimulated for 1 min with increasing concentrations of CXCL12 after addition of coelenterazine 400. Results are expressed as ΔBRET, corresponding to the difference in BRET signal between Gαi-*h*RLuc8 and Gβ_1_γ_2_-GFP10, measured in the presence and absence of CXCL12. The data represent mean values ± SEM (*n* = 6). **(B)** CCR7 changes the basal BRET signal between CXCR4 and G proteins. HEK293T cells were transfected with h*R*Luc-Gαiβγ and CXCR4-Venus in combination or not with CCR7, and interaction between Gαiβγ and CXCR4 was investigated by measuring the energy transfer (BRET^1^) between the partners. The net BRET corresponds to the BRET measured between the two partners minus the BRET measured in cells expressing h*R*Luc-Gαiβγ only. The data represent mean values ± SEM (*n* = 5). **(C)** CCR7 inhibits the CXCL12-induced β-arrestin recruitment to the plasma membrane. HEK293T cells were transfected with the plasma membrane marker K-RasVenus, β-arrestin 2 Luc, and CXCR4 in combination or not with CCR7. The translocation of the β-arrestin 2 to the plasma membrane was recorded after stimulation with increasing concentration of CXCL12 by measuring the energy transfer (BRET) between the BRET energy donor β-arrestin 2 Luc and the BRET energy acceptor K-RasVenus. The data represent mean values ± SEM (*n* = 3).

**Figure 7:**
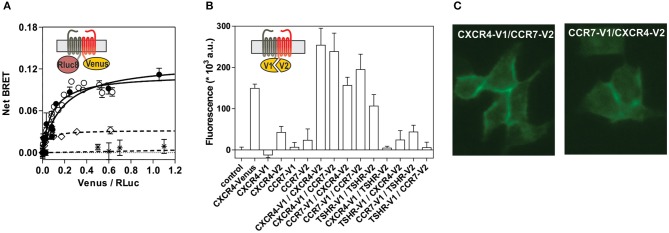
**(A)** CCR7 interacts with CXCR4 in a BRET assay. HEK293T cells were transfected with a constant amount of the CCR7-h*R*Luc fusion and increasing amounts of the CXCR4-Venus fusion (black dots), or a constant amount of the CXCR4-h*R*Luc fusion and increasing amounts of the CCR7-Venus fusion (white dots), and heteromerization of CCR7 and CXCR4 was investigated by measuring the energy transfer (BRET^1^) between the two partners. As a control, an increasing amount of TSHR-Venus was used as BRET acceptor with CXCR4-h*R*Luc (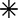) or CCR7-h*R*Luc (♢) as donor. The Net BRET corresponds to the BRET measured between the two partners minus the BRET measured in cells expressing CXCR4-h*R*Luc or CCR7-h*R*Luc only. Data represent mean values ± SEM (*n* = 3). **(B)** CCR7 interacts with CXCR4 in a fluorescence complementation assay. HEK293T cells were transfected with CXCR4-V1, CXCR4-V2, CCR7-V1, and CCR7-V2 constructs, alone or as two by two combinations, and the fluorescence emission was recorded. As controls, TSHR-V1 and TSHR-V2 were cotransfected with the various CXCR4 and CCR7 constructs. Data represent mean values ± SEM (*n* = 3). **(C)** CCR7 interacts with CXCR4 at the plasma membrane. HEK293T cells were cotransfected with CXCR4-V1 and CCR7-V2 or CXCR4-V2 and CCR7-V1, and fluorescence was monitored by using fluorescent microscopy.

**Figure 8:**
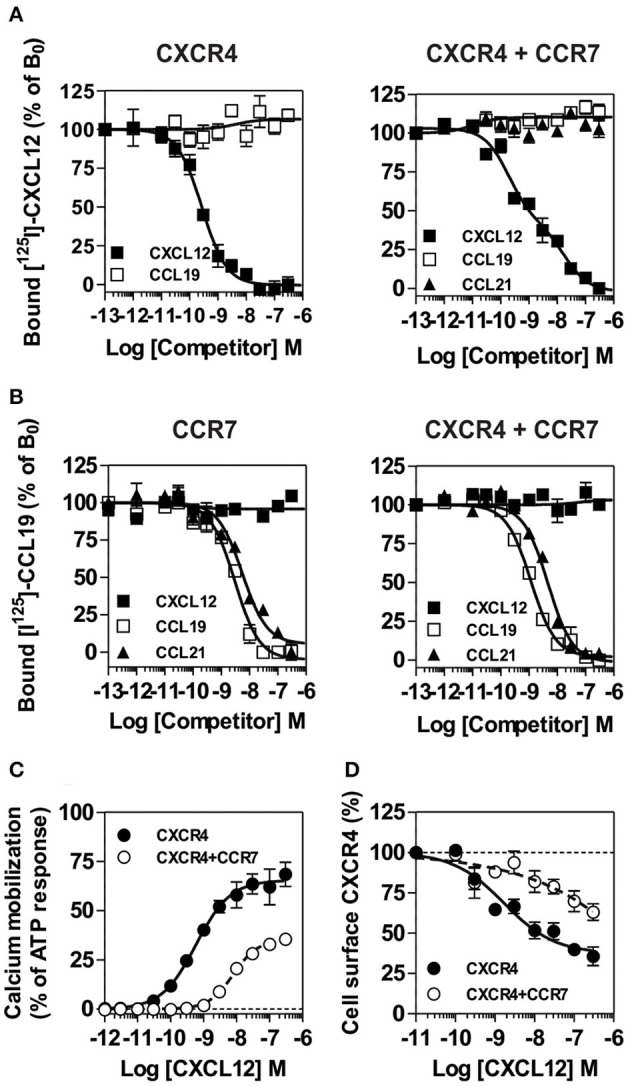
**(A,B)** Competition binding assays in CHO-K1 cells co-expressing CCR7 and CXCR4. Competition binding assays were performed on cells expressing CXCR4 or CCR7 only and on cells co-expressing CXCR4 and CCR7. Cells were incubated with 0.1 nM ^125^I-CXCL12 **(A)** or 0,1nM ^125^I-CCL19 **(B)** as tracers and increasing concentrations of unlabeled CXCL12 (black squares) or CCL19 (white squares) as competitors. The data were normalized for non-specific binding (0%) in the presence of 300 nM of competitor, and specific binding in the absence of competitor (100%). All points were run in triplicates and the data are presented as mean values ± SEM (*n* = 3). **(C)** CCR7 inhibits CXCR4 responsiveness in a calcium mobilization assay. Calcium mobilization assay was performed on CHO-K1 cells stably expressing the calcium-sensitive photoprotein aequorin and CXCR4 only or on cells co-expressing CXCR4 in combination with CCR7. Cells were loaded with coelenterazine H, stimulated with increasing concentrations of CXCL12 and the luminescence was recorded. The results were normalized for baseline activity (0%) and the maximal response obtained with 25 μM ATP (100%). All points were run in triplicates and the data are presented as mean values ± SEM (*n* = 3). **(D)** CCR7 inhibits the downmodulation of CXCR4 induced by CXCL12. CHO-K1 cells expressing CXCR4 only or in combination with CCR7 were either left untreated or stimulated for 90 min with increasing concentrations of CXCL12. Surface-bound CXCL12 was removed by an acid wash step and cell surface expression CXCR4 was estimated by FACS. The data were normalized for the expression of receptor in absence of stimulation (100%). All points were run in duplicates and the data represent mean values ± SEM (*n* = 3).

To better understand the regulation of CXCR4 responsiveness by CCR7, we measured the ability of CXCR4 to activate G proteins using bioluminescence resonance energy transfer (BRET). This technology provides accurate sensitivity to probe the activation of specific G-protein isoforms by measuring the interaction between heterotrimeric Gα*βγ* subunits (24, 26, 27). Cells were transfected with plasmids Gα-*Rluc8*, Gβ, Gγ-GFP10 subunits and the receptors of interest, and the BRET^2^ signal between the BRET donor Gα-*R*Luc8 and the BRET acceptor Gγ-GFP10 was recorded before and after stimulation (Figure 6A). A decrease in the BRET^2^ signal after receptor stimulation reflects the separation between the Gα helical domain and the Gγ N-terminus occurring during GDP-GTP exchange. Analysis show that CXCL12 induced a dose-dependent decrease in the BRET^2^ signal for the three Gαi proteins (Gαi1, Gαi2, and Gαi3) (Figure 6A), while it did not significantly affect the other G proteins (Figure S7). Both the CXCR4 antagonist, AMD3100, and the CXCR4-blocking antibody, MAB173, attenuated the decrease in BRET^2^ signal, demonstrating its specificity (Figure S8). Interestingly, co-expression of CCR7 and CXCR4 resulted in the selective inhibition of Gαi1 and Gαi2 activation by CXCL12, while the profile of Gαi3 activation was insensitive to CCR7 (Figure 6A and Figure S7). Similar to cells expressing CXCR4 only, pretreatment of cells co-expressing CXCR4 and CCR7 with CXCR4-blockers completely inhibited Gαi3 activation, confirming that this signal is dependent on CXCR4 activation (Figure S8). Next, we tested whether the lack of Gαi1 and Gαi2 activation was associated with their inability to interact with CXCR4 in the presence of CCR7. Cells were transfected with plasmids encoding the Gαi-*Rluc8*, Gβ1, Gγ2 subunits and CXCR4-Venus and the BRET^1^ signal between *R*Luc8 and Venus was recorded (Figure 6B). A saturable BRET^1^ signal between the BRET donor Gαi-*RLuc8* and the BRET acceptor CXCR4-Venus when increasing amounts of CXCR4-Venus were coexpressed with a constant amount of Gαi-*Rluc8* reflects the specific interaction between CXCR4 and the G protein. Analysis show that CCR7 coexpression decreased the basal BRET^1^ signal between Gαi1-*RLuc8* and CXCR4-Venus or between Gαi2-*RLuc8* and CXCR4-Venus (Figure 6B). In contrast, the basal BRET^1^ signal between Gαi3-*RLuc8* and CXCR4-Venus was less affected by the presence of CCR7 (Figure 6B). Next, we tested by BRET the ability of CCR7 to interfere with β-arrestin translocation to the plasma membrane. Cells were transfected with plasmids encoding the β-arrestin-Luc, Kras-Venus and the receptors of interest, and the BRET signal between the BRET donor β-arrestin 2-Luc and the BRET acceptor Kras-Venus was recorded before and after stimulation (Figure 6C). In cells expressing CXCR4 only, CXCL12 induces a dose-dependent increase in the BRET signal, reflecting the recruitment of β-arrestin to the plasma membrane. Coexpression of CCR7 and CXCR4 completely abolish the recruitment of β-arrestin, indicating that CCR7 inhibits other pathways (Figure 6C). Collectively, these results may indicate that CCR7 impairs the interaction of CXCR4 with G proteins and β-arrestin. Alternatively, our results could reflect the existence of distinct conformations of CXCR4 signaling complexes resulting from CXCR4 heteromerization with CCR7, as previously reported for other CXCR4 heteromers (28, 29). Thus, we investigated the ability of CXCR4 to interact with CCR7 using a BRET-proximity assay. A specific BRET^1^ signal was detected between CXCR4-*h*RLuc and CCR7-Venus as well as between CCR7-*h*RLuc and CXCR4-Venus, while a much lower BRET^1^ signal was detected between CXCR4-*h*RLuc and the TSHR-Venus used as a specificity control (Figure 7A). Further support for physical and direct interactions between CXCR4 and CCR7 came from bimolecular fluorescence complementation experiments, in which each receptor is fused to complementary fragments of the Venus protein (V1 and V2) (22). Co-expression of CXCR4-V1 and CCR7-V2 generated a significant fluorescent signal at the plasma membrane, confirming the formation of CXCR4/CCR7 heteromers (Figures 7B,C). A much weaker fluorescence was detected in cells expressing the chimera alone or co-expressing the chimera with TSHR-V1 or TSHR-V2. Previously, we showed that heteromerization of CXCR4 with other chemokine receptors results in a strong negative binding cooperativity (22, 23). Using the same methodology, we show here that co-expression of CXCR4 and CCR7 is not associated with negative binding cooperativity. CXCL12 did not inhibit the binding of radiolabeled CCL19 and, conversely, CCL19 and CCL21 did not inhibit the binding of radiolabeled CXCL12 (Figures 8A,B). Nevertheless, the homologous competition performed in cells co-expressing CXCR4 and CCR7 unveiled a second CXCL12 binding site of low affinity (Table S2 and Figure 8A, right panel), which may indicate the presence of a G protein-uncoupled state of CXCR4 within CXCR4/CCR7 heteromers. A second CXCL12 binding site of low affinity is also detected in Nalm6 cells expressing CCR7 (Figure S6). Importantly, we also confirm in CHO-K1 cells that CCR7 inhibited the CXCR4 responsiveness as measured by calcium mobilization and receptor endocytosis assays (Figures 8C,D). Collectively, these results demonstrate that CCR7 interacts with CXCR4 and modifies the conformation of the CXCR4/G protein complexes. It is therefore tempting to link these conformational modifications to the inability of CXCR4 to activate Gαi1 and Gαi2 in the presence of CCR7.

## Discussion

B cells arising from hematopoietic stem cells go through a series of developmental stages in the BM. Soluble factors produced by stromal cells within specific BM niches, such as the chemokine CXCL12, sustain the development and retention of B-cell precursors as they differentiate (30, 31). Ultimately, approximately two-thirds of immature B cells are released into the peripheral blood circulation to reach the spleen and complete their maturation, whereas the remaining B cells mature directly in the BM (32, 33). When pre-B cells differentiate into immature and mature B cells, the responsiveness to CXCR4 decreases dramatically despite the fact that the cell surface expression of the receptor is maintained (15). Such dissociation between CXCR4 expression and responsiveness to CXCL12 has also been reported in other cell types, as well as for other chemokine receptors, but the exact reason for the discrepancy between receptor expression and function often remains undetermined (34–36).

In the present study, we show that the cell surface expression of chemokine receptors changes significantly during B-cell development in the BM. Surface expression of CXCR4 decreases during the transition of pre-B cells to the immature and mature B stages, whereas expression of CCR7 and other chemokine receptors increases. In CCR7-deficient mice, CXCR4 responsiveness is improved in mature B cells, and to a lesser extent in immature B cells. The recovered CXCL12-induced migration in CCR7^−/−^ mature B cells is relatively modest as compared with the migration of pre-B or immature B cells, although the migration index is similar to that triggered by CCL19 or CXCL13. Even though CCR7 regulates CXCR4 responsiveness, the contribution of other factors cannot be formally excluded. Analysis of the ImmGen database (www.Immgen.org) reveals that several genes encoding signaling or regulatory proteins are differentially up- and downregulated between pre-B cells and mature B cells. It has also been reported that the expression of genes encoding signaling proteins and cell adhesion molecules is modulated during B-cell development (37); however, it is difficult to appreciate whether these elements contribute to the regulation of CXCR4 responsiveness.

*In vivo* labeling shows that the proportion of immature and mature B cells located in the parenchyma is higher in CCR7^−/−^ mice, while the number of B cells in sinusoids decreases. These results are in accordance with the role of the CXCL12–CXCR4 axis in the homing of B-cell precursors in the BM, suggesting that CCR7 indirectly regulates the distribution of cells between the two niches (11, 25). Despite a decrease in the egress of immature and mature B cells to the sinusoids of CCR7^−/−^ mice, the number of these cells in the blood is not reduced. These results are in agreement with a previous study showing that CCR7-deficiency does not modify B-cell blood count despite a general defect in the distribution of leukocyte populations among organs (18). We show via adoptive transfer experiments that CXCR4 is key for the short-term homing of immature B cells to the BM, and that CCR7-deficient immature B cells home more readily to the BM. In contrast, CXCL12 is not required for the short-term homing of mature B cells, suggesting that other signals contribute to their homing in the BM. In keeping with this hypothesis, signals mediated by the CB2 receptor have recently been shown to affect the retention of immature and mature B cells in the BM (25). It should also be mentioned that the migration index of mature B cells is relatively low *ex vivo*, suggesting that the CXCL12–CXCR4 axis may efficiently control their retention in the BM while contributing modestly to their recruitment from the bloodstream. Generation of BM chimera shows that the improved retention of B cells in the BM of CCR7^−/−^ mice is not due to an alteration in the BM microenvironment. However, transplantation of BM from CCR7^−/−^ mice into irradiated wild type mice demonstrates that the number of B cells decreases significantly and the relative proportion of the mature B-cell subpopulation increases. These results may indicate that CCR7 is directly involved in hematopoiesis, as has been reported for T lymphocytes (38). Alternatively, a higher number of mature B cells in the BM may also affect the hematopoiesis process through feedback inhibition, as recently suggested (39). Whether CCR7 influences additional B-cell behaviors to retention in the parenchyma remains to be determined.

We also investigated the molecular mechanisms through which CCR7 inhibits CXCR4 responsiveness. Firstly, we show that the inhibition of CXCR4 by CCR7 is selective, since CCR7 is not involved in the regulation of other chemokine receptors expressed by mature B cells. This hypothesis is further supported by previous data showing that CCR7 does not inhibit the function of ChemR23 in recombinant cells, although receptor heteromers were formed (40). Similarly, heteromerization of CXCR4 with other chemokine receptors does not lead to functional inhibition of CXCR4 (22, 23, 40, 41). Therefore, it appears that the functional consequences of receptor co-expression and interaction are highly dependent on the specific pair of receptors considered. Moreover, we demonstrate that CCR7 signaling is not required, since inhibition of CXCR4 function occurred in the absence of CCR7 stimulation. Furthermore, inhibition of CCR7 by blocking antibodies or the use of non-functional CCR7 mutants did not alleviate the inhibition of CXCR4 responsiveness. These findings support a dual role for CCR7 as a signaling receptor and a modulator of CXCR4. The reduced responsiveness of CXCR4 is not due to its inability to interact with CXCL12, since saturation binding assays show that CXCL12 binds with similar efficiency to CXCR4 irrespective of whether CCR7 is expressed. This observation is scarcely compatible with the complete inhibition of interaction with Gαi proteins known to be required for high-affinity binding of chemokines; nevertheless, in competition binding assays, a second low-affinity binding site for CXCL12 was unveiled in cells co-expressing CXCR4 and CCR7. These results may suggest the existence of two CXCR4 populations, one of which displays a reduced propensity to interact with CXCL12. The existence of a CXCR4 population with low binding affinity may account, at least in part, for the decrease in CXCR4 responsiveness detected in native and recombinant cells. It has been reported for some GPCRs that heteromerization modifies their binding properties, and we confirmed such physical interactions between CXCR4 and CCR7. We also demonstrate that CCR7 modifies the CXCR4/Gαi2 complex, either by inhibiting the interaction between CXCR4 and Gαi2 or by changing the conformation of the preformed CXCR4/Gαi2 complex. Using G-protein biosensors, we confirm that the activation of Gαi2 proteins by CXCL12 is also reduced in cells co-expressing CXCR4 and CCR7. The inhibition of Gαi2 activation likely accounts for the decrease in B-cell retention in the BM, since Gαi2 has been shown to be required for CXCR4 signaling in the frame of B-cell chemotaxis (42, 43). It is more difficult to appreciate whether the inhibition of Gαi1 also contributes to the regulation of B-cell retention, since murine lymphocytes express Gαi1 weakly as compared with Gαi2 and Gαi3. Our results also suggest that a functional CXCR4/Gαi3 complex is maintained in the presence of CCR7. Although these results are potentially interesting, it is difficult to confirm, since the precise nature of the signals downstream of Gαi3 is not known (44); thus, whether Gαi3-dependent signaling still exists in cells co-expressing CXCR4 and CCR7 remains an open question that requires further analysis.

In the present study, we show that CXCR4–CCR7 heteromerization results in deficient activation of Gαi1 and Gαi2 proteins. The upregulation of CCR7 and the concomitant decrease in CXCR4 during B-cell development may favor the formation of heteromers, leading to an eventual defect in Gαi1/2 signaling. It has been previously shown that CXCR4 can interact with several other GPCRs including the chemokine receptors CCR2, CCR5, and CXCR7, the Epstein–Barr Virus-encoded BILF1, and the β2-adrenergic receptor. Heteromerization of CXCR4 with CCR2 and CCR5 results in a strong negative binding cooperativity of an allosteric nature i.e., the specific ligands for CCR2 and CCR5 inhibit the binding of CXCL12 to CXCR4 (22, 23). The co-expression of CXCR7 and CXCR4 has been reported to inhibit the activation of Gαi proteins by CXCR4 (28); however, whether this effect requires CXCR4 heteromerization remains to be determined, since CXCR7 can also bind CXCL12 with high affinity and promote a range of cellular responses (45, 46). In another study, it was reported that the co-expression of CXCR7 and CXCR4 results in the constitutive recruitment of β-arrestin-2 to CXCR4–CXCR7 heteromers and subsequent enhancement of cell migration (47). Co-expression of BILF1 and CXCR4 has been shown to almost completely inhibit CXCL12 binding to CXCR4 as a consequence of BILF1 constitutive activity; however, it is unknown whether this effect is linked to CXCR4/BILF1 heteromerization (48). Finally, β2-adrenergic receptor activation has been reported to enhance CXCR4 signaling, promoting the retention of T lymphocytes in lymph nodes, but whether this increase in CXCR4 responsiveness is caused by its physical interaction with β2 adrenergic receptor remains to be established (49).

Our findings reveal the existence of a novel regulatory mechanism of CXCR4 function, which, to the best of our knowledge, is the first indication that chemokine receptor co-expression has consequences for a physiological process. Our results may also have important implications in pathological contexts. Several studies have highlighted the critical role of the CXCL12–CXCR4 axis in cancer progression, and overexpression of CXCR4 by tumor cells is generally considered a poor prognostic marker. Yet, a lack of correlation between CXCR4 expression and cell migration has been reported in some studies (50, 51). The expression of accessory proteins, such as ZAP70 or CXCR7, in tumors has been shown to regulate the function of CXCR4 (52, 53). Heteromerization of CXCR4 and CCR7 in certain tumors may be an alternative mechanism to explain the lack of correlation between CXCR4 expression and responsiveness. Our discovery that CXCR4 function *In vivo* depends on the expression of another chemokine receptor adds a new dimension to our understanding of the manner by which CXCR4 may control cell behavior in physiological and pathological processes.

## Data Availability Statement

The raw data supporting the conclusions of this article will be made available by the authors, without undue reservation, to any qualified researcher.

## Ethics Statement

Animal experimentation was carried out in accordance with European (EU Directives 86/609/EEC) and national guidelines. All procedures were reviewed and approved by the local ethical committee (Commission d'Ethique du Bien-Etre Animal, CEBEA) of the Université Libre de Bruxelles.

## Author Contributions

SM, NV, CD, CG, and J-YS performed experiments, analyzed the data, and generated figures. J-YS designed experiments. J-YS and MP supervised experiments and analysis. J-YS and MP wrote the manuscript with contributions from the other authors.

### Conflict of Interest

The authors declare that the research was conducted in the absence of any commercial or financial relationships that could be construed as a potential conflict of interest.
