# Supplementary material for: Coexpression of CCR7 and CXCR4 During B Cell Development Controls CXCR4 Responsiveness and Bone Marrow Homing
Source: Front Immunol. 2019 Dec 18;10:2970. doi: 10.3389/fimmu.2019.02970 (PMC6930800; doi:10.3389/fimmu.2019.02970)
Supplement: Supplementary file 1 [file Data_Sheet_1.PDF]

Table S1. Plasmids used in this study			
**Plasmids**	**Modifications**	**Position**	**Reference**
pcDNA3-CXCR4	-	-	This study
pcDNA3-CCR7	-	-	This study
pcDNA3-CXCR4-Ruc8	RLuc8	CT	Sohy et al., 2009
pcDNA3-CXCR4-Venus	Venus	CT	Sohy et al., 2009
pcDNA4-CCR7-RLuc8	RLuc8	CT	This study
pcDNA3-CCR7-Venus	Venus	CT	This study
pcDNA3-TSHR-Venus	Venus	CT	This study
pcDNA3-CXCR4-V1	Venus 1-156	CT	Sohy et al., 2009
pcDNA3-CXCR4-V2	Venus 157-239	CT	Sohy et al., 2009
pcDNA3-CCR7-V1	Venus 1-156	CT	This study
pcDNA3-CCR7-V2	Venus 157-239	CT	This study
pcDNA3-TSHR-V1	Venus 1-156	CT	Sohy et al., 2009
pcDNA3-TSHRV2	Venus 157-239	CT	Sohy et al., 2009
pcDNA3-Gαi1	-	-	cDNA Resource Center (www.cDNA.org)
pcDNA3-Gαi2	-	-	cDNA Resource Center (www.cDNA.org)
pcDNA3-Gαi3	-	-	cDNA Resource Center (www.cDNA.org)
pcDNA3-Gβ1	-	-	Saulière et al., 2012
pcDNA3-γ2	-	-	cDNA Resource Center (www.cDNA.org)
pcDNA3-Gαi1-RLuc8	RLuc8	91-92	Saulière et al., 2012
pcDNA3-Gαi2-RLuc8	RLuc8	91-92	Saulière et al., 2012
pcDNA3-Gαi3-RLuc8	RLuc8	91-92	Saulière et al., 2012
pcDNA3-Gαoa-RLuc8	RLuc8	91-92	Saulière et al., 2012
pcDNA3-Gαob-RLuc8	RLuc8	91-92	Saulière et al., 2012
pcDNA3-Gαs-RLuc8	RLuc8	113-114	Saulière et al., 2012
pcDNA3-Gαq-RLuc8	RLuc8	97-98	Saulière et al., 2012
pcDNA3-Gα11-RLuc8	RLuc8	97-98	Saulière et al., 2012
pcDNA3-Gα12-RLuc8	RLuc8	115-116	Saulière et al., 2012
pcDNA3-Gα13-RLuc8	RLuc8	106-107	Saulière et al., 2012
pcDNA3-Gγ2-GFP10	GFP10	NT	Saulière et al., 2012
pcDNA3-Arrestin-Luc	Luc	NT	Corbisier et al 2015
pcDNA3-KrasVenus			Lan et al., 2012
pEFIN3-CCR7	-	-	De Poorter et al., 2013
pEFIN3-HACCR7ΔNT	(1) HA-Tag<br>(2) D/A and E/A	(1) NT<br>(2) D2; D26; E27;D30-31; D35; D40; E45; D52	This study
pEFIN3-CCR7(N/A)PXXY	N/A	N322	This study
pEFIN3-CCR7D(R/A)Y	R/A	R154	This study
pEFIN3-CCR5	-	-	El-Asmar et al., 2005

NT : N-terminus ; CT :C-terminus
