# Supplementary material for: Coexpression of CCR7 and CXCR4 During B Cell Development Controls CXCR4 Responsiveness and Bone Marrow Homing
Source: Front Immunol. 2019 Dec 18;10:2970. doi: 10.3389/fimmu.2019.02970 (PMC6930800; doi:10.3389/fimmu.2019.02970)
Supplement: Supplementary file 2 [file Data_Sheet_2.PDF]

Table S2, Binding parameters of CHO-K1 cells expressing CXCR4 and CCR7

Cells	Tracer	Competitor	IC_{50} (nM)	% of inhibition
CXCR4	[^{125}I]-CXCL12	CXCL12	0.80 ± 0.05	100
CCR7	[^{125}I]-CCL19	CCL19	1.84 ± 1.18	100
		CCL21	4.06 ± 1.68	96.5 ± 6.5
CXCR4 + CCR7	[^{125}I]-CXCL12	CXCL12	(a) 0.19 ± 0.01<br>(b) 30.88 ± 12.52	100
		CCL19	n.d.	n.d.
		CCL21	n.d.	n.d.
	[^{125}I]-CCL19	CXCL12	n.d.	n.d.
		CCL19	1.70 ± 0.46	100
		CCL21	4.20 ± 0.50	98.5 ± 3.5

Binding parameters were measured on cells expressing CXCR4 and CCR7. The IC_{50} and % of inhibition values were obtained from competition binding experiments as displayed in Fig. 8. Values represent the mean ± S.E.M. of at least three independent experiments. n.d. not detected.
