# Supplementary material for: Coexpression of CCR7 and CXCR4 During B Cell Development Controls CXCR4 Responsiveness and Bone Marrow Homing
Source: Front Immunol. 2019 Dec 18;10:2970. doi: 10.3389/fimmu.2019.02970 (PMC6930800; doi:10.3389/fimmu.2019.02970)
Supplement: Supplementary file 3 [file Data_Sheet_3.PDF]

Supplementary Figures

Mcheik et al.

Fig. S1 Identification of B cell subpopulations.

Fig. S2 Alternative staining procedure to discriminate B cell subpopulations and chemotaxis of B cell subsets.

Fig. S3 B cell subsets in bone marrow and blood.

Fig. S4 B cell subsets in bone marrow and blood identified by the alternative staining procedure.

Fig. S5 CCR7 deficiency does not increase the number of T cells in bone marrow.

Fig. S6 Expression of CCR7 on Nal6 cells

Fig. S7 Panel of G proteins activated by CXCR4.

Fig. S8 CXCR4 antagonists inhibit G protein activation.

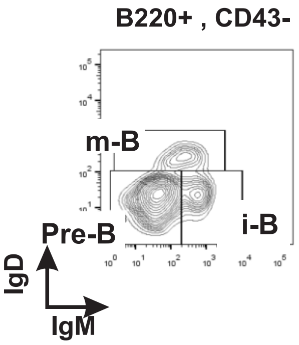

Figure S1. Identification of B cell subpopulations. Bone marrow B cell subpopulations were discriminated by flow cytometry using AF700-conjugated anti-B220, FITC-conjugated anti-CD43, PerCP-Cy5.5-conjugated anti-IgM and V450-conjugated anti-IgD antibodies. Pro/Pre-B cells were identified as $B220^{+}/CD43^{-}/IgM^{-}/IgD^{-}$, immature B cells as $B220^{+}/CD43^{-}/IgM^{+}/IgD^{-}$ and mature B cells as $B220^{+}/CD43^{-}/IgM^{+}/IgD^{+}$. A density plot representative of B cell populations from of a $CCR7^{+/+}$ mouse is shown.

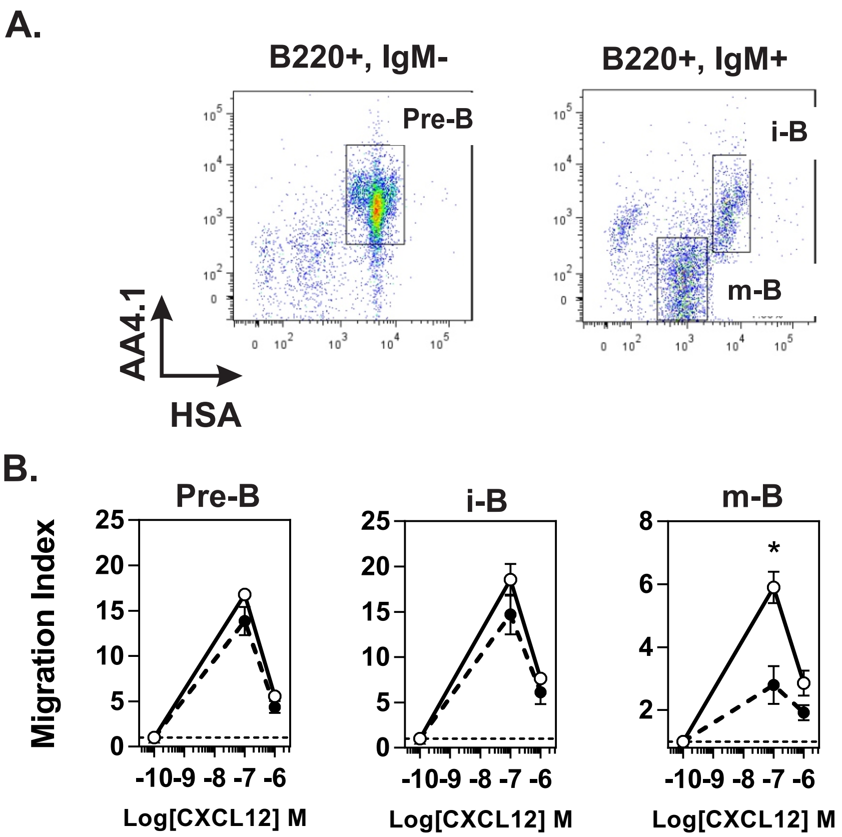

Figure S2 A. Alternative staining procedure to discriminate B cell subsets. Bone marrow B cells were discriminated by flow cytometry using AF700-conjugated anti-B220, PercP-Cy5/5-conjugated IgM, PE-conjugated AA4.1 and FITC-conjugated HSA (CD24) antibodies. Pre-B cells were identified as $B220^+$, IgM^-, $AA4.1^+$, HSA^+, immature B cells as $B220^+$, IgM^+, $AA4.1^+$, HSA^+ and mature B cells as $B220^+$, IgM^+, AA4.1 low, HSA low. **B. Chemotaxis of B cells subsets.** Transwell migration of bone marrow cells from $CCR7^{+/+}$ (black dots) or $CCR7^{-/-}$ (white dots) mice in response to 100nM an 1µM CXCL12. All conditions were run in triplicated and the data are presented as mean ± SEM, * $P<0.05$.

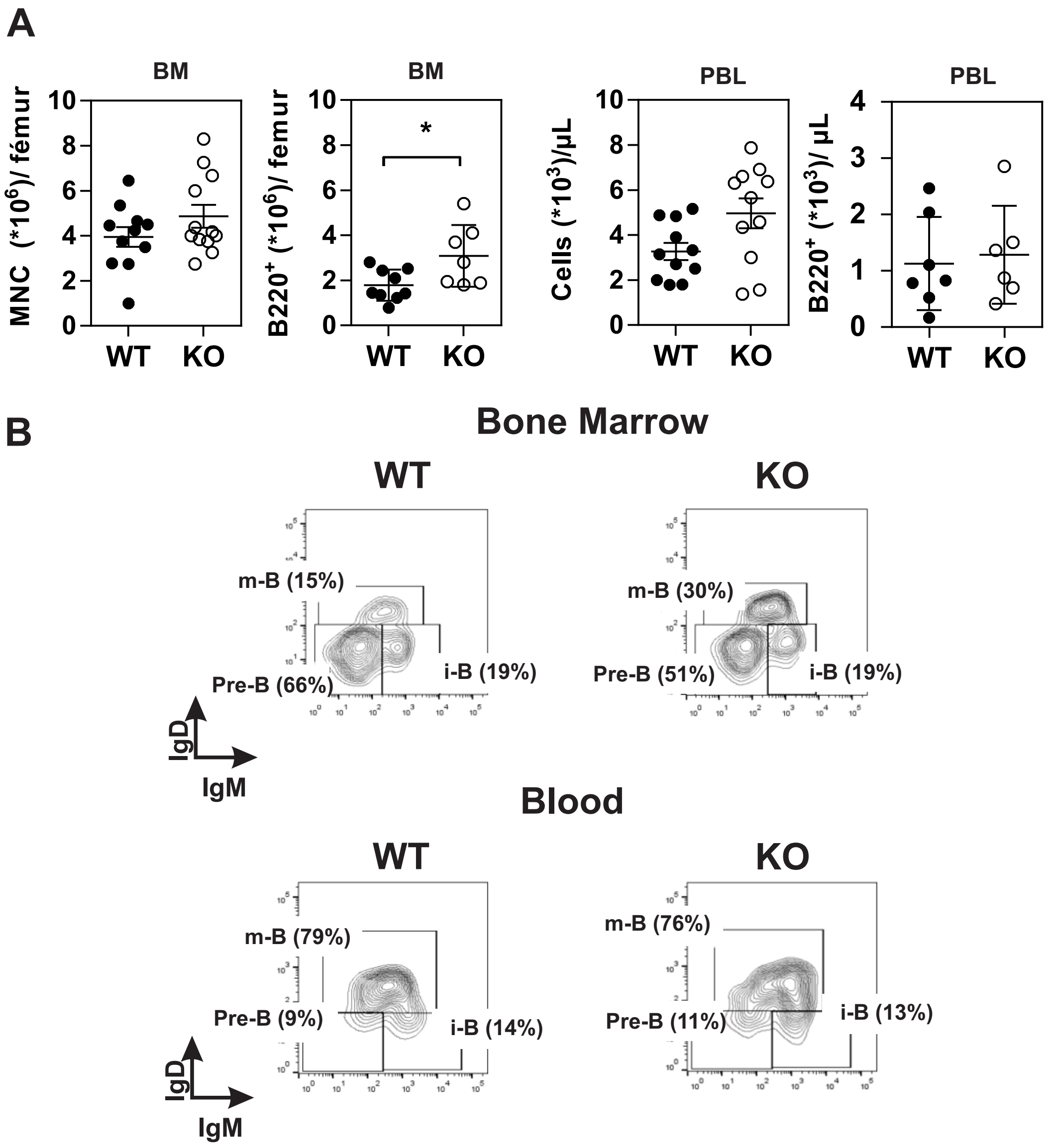

Figure S3 B cells in bone marrow and blood A. Increase number of B cells in the bone marrow of CCR7$^{-/-}$ mice. Number of mononuclear (MCN) and B (B220^{+}) cells in the bone marrow of wild type (black dots) and CCR7$^{-/-}$ mice (white dots). Data are represented as the mean values ± SEM and each dot corresponds to individual mice (n= 10-13 mice, *, P<0.05). **B.** Representative flow cytometry plots of B cell subpopulations in bone marrow and blood of CCR7$^{+/+}$ and CCR7$^{-/-}$ mice. Pre-B cells (B220^{+}, CD43^{-}, IgM^{-}, IgD^{-}), immature B cells as (B220^{+}, CD43^{-}, IgM^{+}, IgD^{-}) and mature B cells (B220^{+}, CD43^{-}, IgM^{+}, IgD^{+}). Of note, caution should be taken regarding the proportion of Pre-B and i-B in blood as this labelling strategy poorly discriminates these populations.

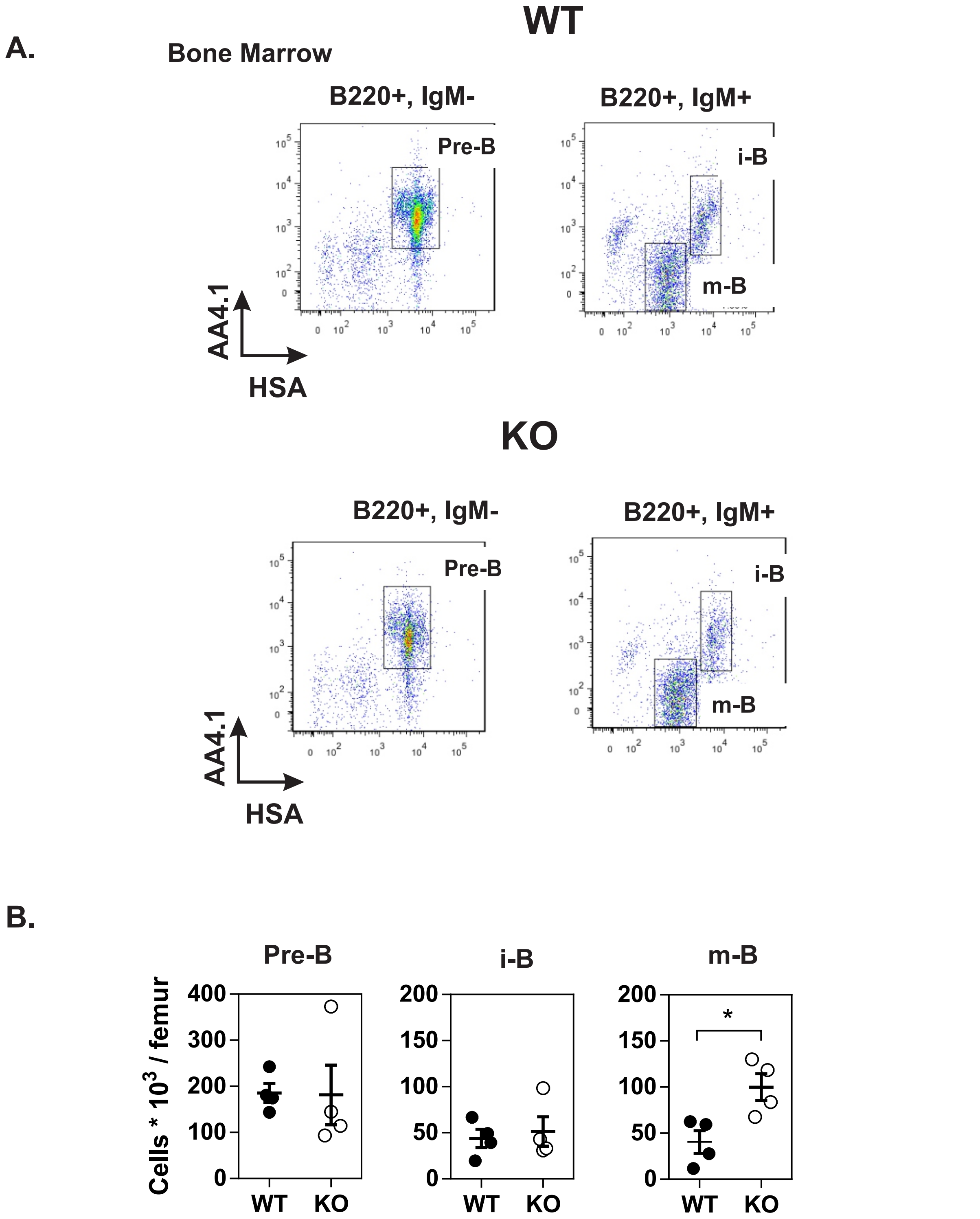

Figure S4 A. B cell subsets in bone marrow identified by the alternative staining procedure. A. Representative flow cytometry plots of bone marrow B cell subpopulations of CCR7$^{+/+}$ and CCR7$^{-/-}$ mice. Pre-B (B220^{+}, IgM^{-}, AA4.1^{+}, HSA^{+}), immature B cells (B220^{+}, IgM^{+}, AA4.1^{+}, HSA^{+}) and mature B cells (B220^{+}, IgM^{+}, AA4.1 ^{low}, HSA^{low}). **B. Number of B cells in the bone marrow.** The number of B cell subpopulations in the bone marrow of CCR7$^{+/+}$ (black dots) or CCR7$^{-/-}$ (white dots) mice was analyzed by flow cytometry. Data are presented as mean value ± SEM and each dots correspond to individual mice (n=4 mice, * P<0.05).

Bone Marrow

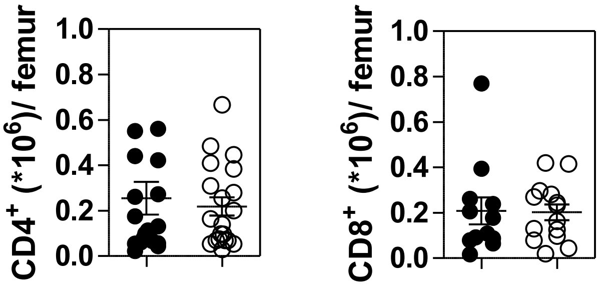

Figure S5. CCR7 deficiency does not increase the amount of T cells in bone marrow. $CD4^+$ and $CD8^+$ T cells from the bone marrow of $CCR7^{+/+}$ (black dots) and $CCR7^{-/-}$ (white dots) mice were analyzed by flow cytometry. Data are represented as mean values ± SEM where each dot represents individual mice (n= 9 to 16 mice per group).

A.

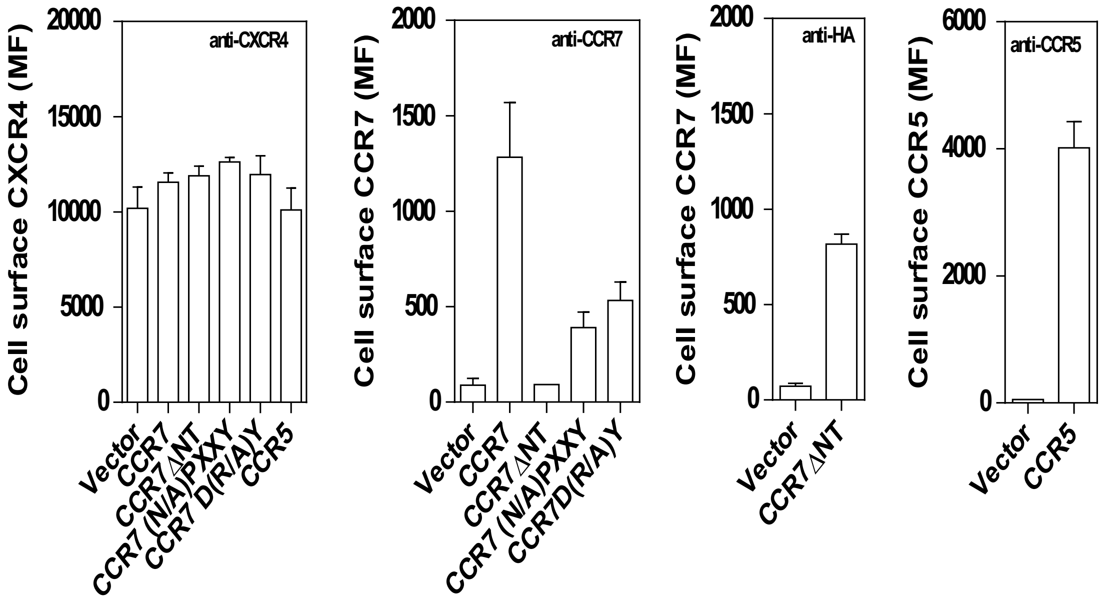

B.

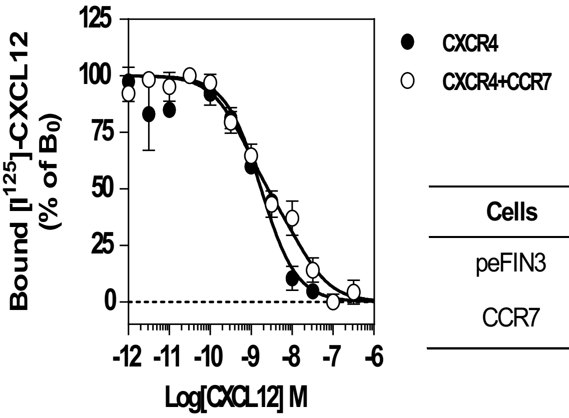

Cells	Tracer	Competitor	IC_{50} (nM)
peFIN3	$[^{125}I]$-CXCL12	CXCL12	1,7 ± 0,05
CCR7	$[^{125}I]$-CXCL12	CXCL12	(a) 0,66 ± 0,01<br>(b) 15,56 ± 12,52

Figure S6 A. Expression of CCR7 mutants does not inhibit CXCR4 expression at the cell surface. Nalm-6 cells were stably transfected with plasmids encoding wild type CCR7, CCR7 mutants and CCR5 as control, and cell surface expression of CXCR4, CCR7 and CCR5 was monitored by flow cytometry. Since the CCR7ΔNT is not detected by the anti-CCR7 antibody, an additional HA-tag was added at the N-terminus and the expression at the cell surface of this mutant was monitored by using anti-HA antibody. The data represent mean values of the mean fluorescent index ± SEM. B. Competition binding assay in Nalm6 cells expressing CCR7. Competition binding assay was performed on Nalm-6 cells transfected with vector only (Black dots) or Nalm-6 cells transfected with CCR7 (White dots). Cells were incubated with 0,1 nM ^{125}I-CXCL12 as tracer and increasing concentrations of unlabeled CXCL12 as competitor. The data were normalized for non-specific binding (0%) in the presence of 300 nM CXCL12 and specific binding (100%) in the absence of competitor. The data represent the mean values ± SEM.

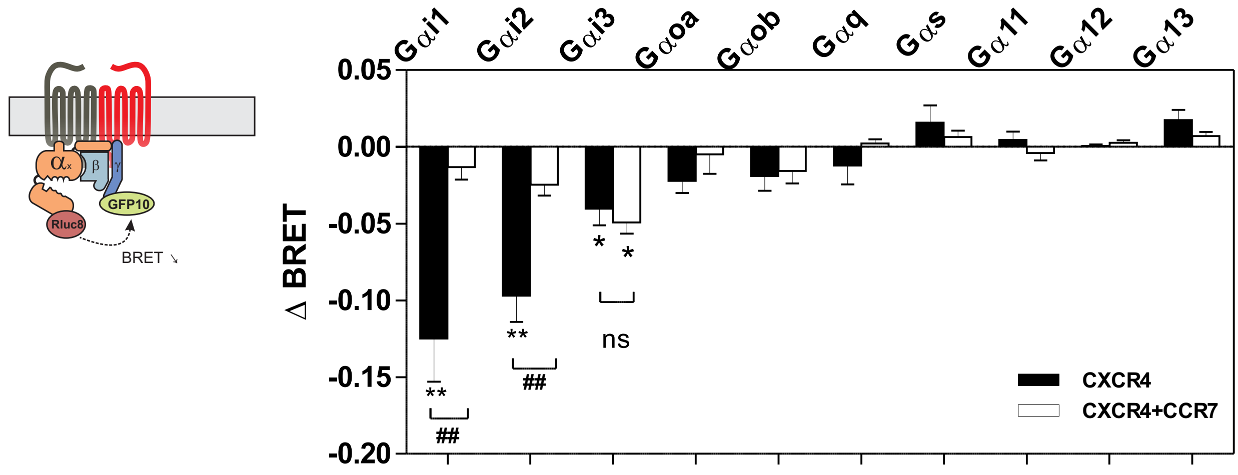

Figure S7. Panel of Gα proteins activated by CXCR4. Real-time measurement of BRET signal in HEK293T cells coexpressing various Gα biosensors with CXCR4 only (black bars) or in combination with CCR7 (white bars). Cells were stimulated one minute with 100 nM CXCL12 after addition of coelenterazine 400. Results are expressed as ΔBRET corresponding to the difference in BRET signal between Gαi-*h*RLuc8 and $G\beta_1\gamma_2$-GFP10 measured in the presence and absence of CXCL12. Data are represented as mean values ± SEM (n= 6 , *** P<0,0005).

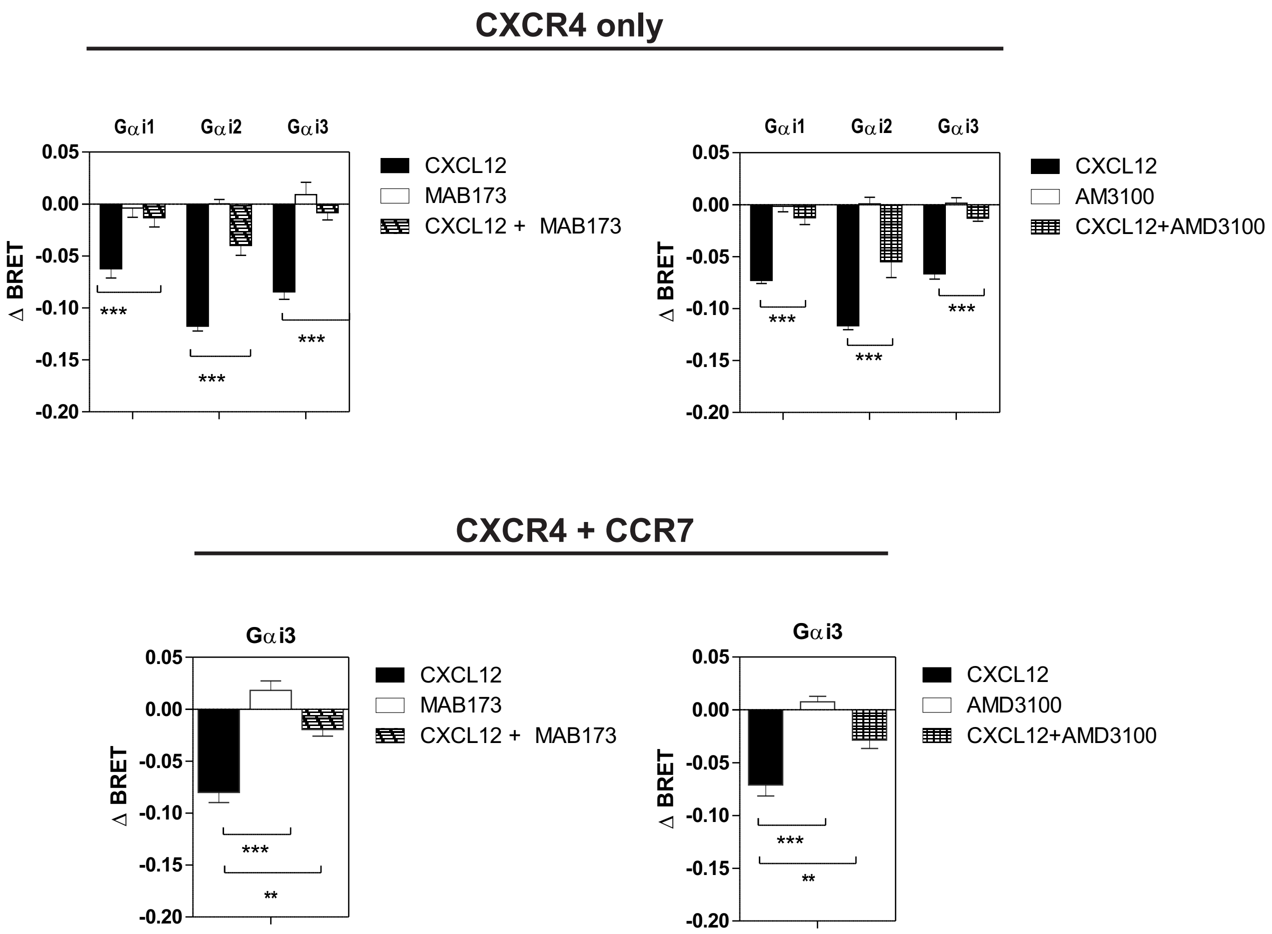

Figure S8. Activation of Gα proteins by CXCL12 is inhibited by CXCR4 antagonists. Real-time measurement of BRET signal in HEK293T cells coexpressing various Gαi biosensors with CXCR4 only (top) or in a combination with CCR7 (bottom). Cells were either left untreated or incubated 20 minutes with the anti-CXCR4 antibody MAB173 or the small molecule antagonist AMD3100 prior stimulation with 25 nM CXCL12. Results are expressed as ΔBRET corresponding to the difference in BRET signal between Gαi-*h*RLuc8 and $G\beta_1\gamma_2$-GFP10 measured in the presence and absence of CXCL12. Data are presented as mean values ± SEM (n=6; ** P<0.005, *** P<0,0005).
